# AN INNOVATIVE MODEL OF CARE FOR SUBJECTS WITH DEMENTIA IN ITALY—IL PAESE RITROVATO—THROUGH THE SARS-COV-2 PANDEMIC

**DOI:** 10.1093/geroni/igac059.1613

**Published:** 2022-12-20

**Authors:** Paolo Mazzola, Gaia Ferraguzzi, Mariella Zanetti, Giuseppe Bellelli

**Affiliations:** Università degli Studi di Milano-Bicocca, Monza, Lombardia, Italy; Università degli Studi di Milano, Milano, Lombardia, Italy; Cooperativa La Meridiana, Monza, Lombardia, Italy; University of Milano-Bicocca, Monza, Lombardia, Italy

## Abstract

“Il Paese Ritrovato” is an innovative long-term facility dedicated to subjects with mild-to-moderate Alzheimer’s disease or other dementias, located in Monza (Lombardia, Italy).By living in a village that resembles a community neighborhood, it aims at people’s wellbeing and represents a shift in the paradigm of traditional nursing home care, allowing the guests to visit the hair stylist, the supermarket, and various activities according to their personal habits, always supported by trained professionals.We performed a retrospective cohort study on 60 subjects, comparing their characteristics before and during the SARS-CoV-2 pandemic, from June 2018 to December 2020. We collected demographic information, cognition, comorbidity, functional status, engagement in the activities through Popularity Index (PI) and Engagement Social Index (ISE), and use of psychoactive medications.The follow-up is performed every 6 months from admission: T0, T6, T12, and T18 are the time-points. The assessment performed during the pandemic is called Tcovid.Compared to a relative stability in cognition before the pandemic, the assessment at Tcovid showed an accelerated worsening of MMSE and CDR scales. We observed an increase in the prescription of antipsychotics (+5%), antidepressants (+11.7%), and benzodiazepines (+1.7%). As expected, engagement in activities dropped (lower PI and ISE). Functional status gradually worsened during the follow-up, according to the natural progression of dementias, but Tcovid slightly accelerated this process and overall worsened the balance performances.Although we are currently unable to quantify the negative effect of this reorganization, future studies need to address the real impact of pandemic on the guests’ performances.

